# Pancreatic Adenocarcinoma Producing Parathyroid Hormone-Related Protein

**DOI:** 10.1155/2017/5656130

**Published:** 2017-08-15

**Authors:** Reiko Yamada, Kyosuke Tanaka, Hiroyuki Inoue, Takashi Sakuno, Tetsuro Harada, Naohiko Yoshizawa, Hiroshi Miura, Toshihumi Takeuchi, Misaki Nakamura, Masaki Katsurahara, Yasuhiko Hamada, Noriyuki Horiki, Yoshiyuki Takei

**Affiliations:** ^1^Department of Gastroenterology and Hepatology, Mie University Graduate School of Medicine, Tsu, Japan; ^2^Department of Endoscopy, Mie University School of Medicine, Tsu, Japan

## Abstract

A 48-year-old woman presented to our hospital with a 1-year history of a continuous high fever. She was diagnosed with metastatic pancreatic adenocarcinoma accompanied by leukocytosis without infection. Her serum concentration of granulocyte colony-stimulating factor was highly elevated. Forty-five days after initiating chemotherapy, she was readmitted because of a neuropsychiatric disturbance and hypercalcemia. Her serum concentration of parathyroid hormone-related protein (PTH-rP) was elevated. A pretreatment biopsy specimen showed strong cytoplasmic immunoreactivity to anti-PTH-rP antibody, suggesting that overproduction of PTH-rP accounted for the hypercalcemia. Although the patient regained consciousness after treatment, she died of progressive disease 60 days after chemotherapy.

## 1. Introduction

Paraneoplastic syndromes are sometimes seen in patients with advanced malignancies. Tumors that produce parathyroid hormone-related protein (PTH-rP) can cause a paraneoplastic syndrome characterized by hypercalcemia [1]. The PTH-rP concentration is elevated in more than 90% of patients with squamous cell, renal, ovarian, breast, and endometrial cancer or human T-lymphotropic virus-associated lymphoma [1, 2]; however, it is rarely elevated in patients with pancreatic adenocarcinoma [3–6]. Hence, paraneoplastic production of PTH-rP by pancreatic adenocarcinomas is highly unlikely. We herein report a rare case of a pancreatic adenocarcinoma that produced PTH-rP, resulting in hypercalcemia.

## 2. Case Report 

A 48-year-old woman was referred to our hospital with a 1-year history of a continuous high fever. Significant events in her medical history included Graves' disease at 39 years of age and the removal of an ovarian cyst at 45 years of age. At the first time when she presented to the referring hospital with a high fever, computed tomography (CT) showed no apparent lesion. After surveillance, her fever was initially thought to be due to tonsillitis. A tonsillectomy was performed at the referring hospital; however, the fever persisted. Follow-up CT revealed a pancreatic body mass and multiple liver masses. Based on these findings, the patient was subsequently referred to our institution for further examination.

**Figure 1:**
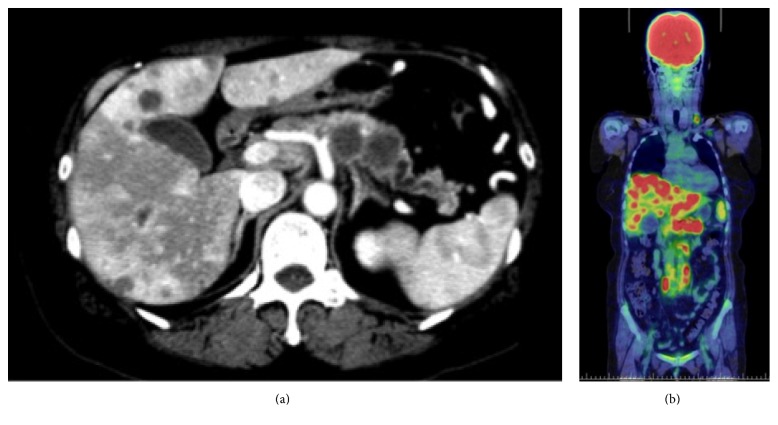
Computed tomography findings. (a) Contrast-enhanced computed tomography showed a hypovascular tumor in the pancreatic body and multiple liver masses with marginal enhancement. (b) Positron emission tomography-computed tomography showed accumulation of ^18^F-fluorodeoxyglucose in the pancreatic body mass [maximum standardized uptake value (SUV max), 7.6], liver masses (SUV max, 9.4), swollen lymph nodes (SUV max, 8.4), and lung masses (SUV max, 1.0).

Initial laboratory tests showed leukocytosis (white blood cell count, 17,930/mm^3^) and an elevated serum C-reactive protein (CRP) concentration (16.79 mg/dL) (Table 1). Blood cultures taken several times showed no signs of infection. Contrast-enhanced CT revealed a pancreatic body tumor with a diameter of 42 mm (Figure 1(a)) and multiple liver masses with marginal enhancement. ^18^F-Fluorodeoxyglucose (^18^F-FDG) positron emission tomography combined with CT showed ^18^F-FDG accumulation in the pancreatic mass [maximum standardized uptake value (SUV max), 7.6], liver masses (SUV max, 9.4), swollen lymph nodes (SUV max, 8.4), and several lung masses (SUV max, 1.0); however, there were no signs of bone metastasis (Figure 1(b)).

**Figure 2:**
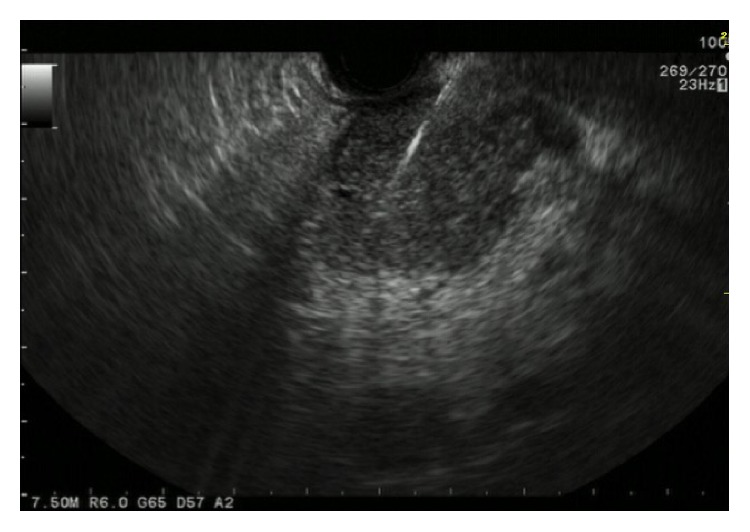
Endoscopic ultrasound findings. A dumbbell-shaped mass was present in the body of the pancreas.

**Figure 3:**
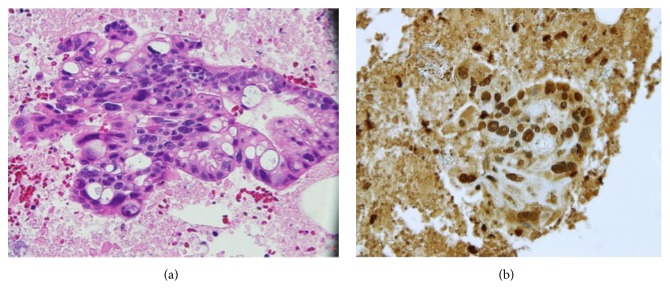
Pathologic examination findings. (a) Examination of an endoscopic ultrasound-guided fine-needle aspiration specimen showed necrotic tissue and tumor cells with highly atypical nuclei. Based on the histological findings, the final diagnosis was pancreatic adenocarcinoma. (b) Immunohistochemical staining of an endoscopic ultrasound-guided fine-needle aspiration specimen showed strong expression of parathyroid hormone-related protein.

To obtain a definitive diagnosis, endoscopic ultrasound-guided fine-needle aspiration biopsy (EUS-FNAB) was performed using a 22-gauge FNA needle (EchoTip; Cook Medical, Bloomington, IN) (Figure 2). EUS revealed a dumbbell-shaped mass in the body of the pancreas. Histopathologic examination of the biopsy specimen showed necrotic tissue and tumor cells with highly atypical nuclei (Figure 3(a)), indicating a diagnosis of pancreatic adenocarcinoma. Tumor fever was the most likely cause of fever, because several blood cultures showed no signs of infection. The serum granulocyte colony-stimulating factor (G-CSF) concentration was 85.1 pg/mL (normal range, <39 pg/mL); however, immunohistochemical (IHC) staining of the EUS-FNAB specimen for G-CSF showed demonstrated negative findings.

Oral administration chemotherapy of S-1, which is an oral fluoropyrimidine preparation, at 30 mg/m^2^ twice daily on days 1 to 28 of each 42-day cycle was initiated. Although the treatment had been progressing without side effect, the patient was urgently admitted to the hospital 45 days after the commencement of chemotherapy because of neuropsychiatric symptoms during a scheduled follow-up visit; she had hallucinations and was wandering in her house at the day before admission. On admission, she was confused but able to localize pain and open her eyes in response to call.

Hematologic examination showed marked leukocytosis (white blood cell count, 58,540/mm^3^), an elevated CRP concentration (13.80 mg/dL), and severe hypercalcemia (calcium concentration, 18.7 mg/dL, corrected for the albumin of 2 mg/dL) (Table 1). In addition, the serum PTH-rP concentration was 13.7 pmol/mL, which well exceeded the normal range (<1.1 pmol/mL). Production of PTH-rP by the tumor was confirmed via IHC staining of the pretreatment EUS-FNAB specimen previously obtained; strong expression of PTH-rP in the tissue of pancreatic adenocarcinoma was observed (Figure 3(b)). Hence, the hypercalcemia was likely caused by tumor-derived PTH-rP.

Parenteral hydration combined with furosemide was performed, followed by administration of synthetic calcitonin and bisphosphonate. The patient regained consciousness, and her serum calcium level normalized to 9.0 mg/dL. She was communicative and in good spirits for several days thereafter. However, she ultimately died of progressive disease 60 days after the commencement of chemotherapy.

## 3. Discussion

In the present case, the serum PTH-rP and G-CSF concentrations were markedly elevated in a patient with a pancreatic adenocarcinoma. The strong expression of PTH-rP in the tissue of pancreatic adenocarcinoma was observed; thus production of PTH-rP by the tumor was confirmed, whereas IHC staining for G-CSF was negative in spite of markedly elevated serum G-CSF concentration. Hence, G-CSF elevation might occur secondary to inflammation.

PTH-rP is a single-chain peptide with an amino terminal domain that is very similar to that of PTH. PTH-rP is also known as an oncofetal protein expressed in both normal tissues and many malignancies, including squamous cell, renal, ovarian, breast, and endometrial cancers; however, it is rarely elevated in patients with pancreatic adenocarcinoma [2–6]. PTH-rP seems to play a role in cell growth, proliferation, and angiogenesis [7]. By interacting with its classic bone and kidney receptors, excess PTH-rP triggers an endocrine response that results in hypercalcemia.

Our patient exhibited an attenuated inflammatory reaction along with a continuous high fever and high serum CRP concentration. These findings suggest that inflammatory cytokines played a key role in the production of PTH-rP by cancer cells and the high serum G-CSF concentration. An association between hypercalcemia and inflammatory cytokines has been reported [8–10]. Our findings suggest that continuous inflammation in association with rapid tumor growth may exacerbate the severe hypercalcemia caused by excess PTH-rP, ultimately resulting in the poor prognosis of pancreatic adenocarcinomas. However, further in vitro and in vivo studies are needed to confirm this theory in patients with pancreatic adenocarcinomas.

In conclusion, if a patient with pancreatic adenocarcinoma becomes an abnormal neuropsychiatric condition, hypercalcemia due to PTH-rP production by cancer cells should also be considered.

## Figures and Tables

**Table 1:** Laboratory findings on first admission and second admission.

Laboratory data on first admission		Laboratory data on second admission	
WBC	17.930/*μ*L	WBC	58.540/*μ*L
Seg + band	85.7%	Seg + band	96.0%
Lymph.	6.6%	Lymph.	1.5%
Mono.	7.4%	Mono.	2.0%
Eosin.	0.2%	Eosin.	0.5%
Baso.	0.1%	Baso.	0%
RBC	4.06 × 10^6^/*μ*L	RBC	2.74 × 10^6^/*μ*L
Hb	10.3 g/dL	Hb	8.8 g/dL
Ht	32.0%	Ht	27.3%
Plt	352 × 10^3^/*μ*L	Plt	329 × 10^3^/*μ*L
APTT	38.6 sec	APTT	35.0 sec
PT	13.2 sec	PT	17.2 sec
Alb	3.4 g/dL	Alb	2.0 g/dL
T-bil	0.7 mg/dL	T-bil	2.7 mg/dL
AST	29 U/L	AST	82 U/L
ALT	31 U/L	ALT	47 U/L
LDH	215 U/L	LDH	468 U/L
ALP	669 U/L	ALP	1263 U/L
*γ*-GTP	127 U/L	*γ*-GTP	234 U/L
BUN	6 mg/dL	BUN	36 mg/dL
Cre	0.44 mg/dL	Cre	0.85 mg/dL
Glu	117 mg/dL	Glu	117 mg/dL
CRP	16.79 mg/dL	CRP	13.80 mg/dL
Na	130 mmol/L	Na	130 mmol/L
K	3.5 mmol/L	K	4.0 mmol/L
Cl	99 mmol/L	Cl	99 mmol/L
Ca	9.4 mg/dL	Ca	17.1 mg/dL
P	3.5 mg/dL	P	3.9 mg/dL
CEA	5.7 ng/mL	CEA	21.9 ng/mL
CA19-9	1.0 U/mL	CA19-9	1.0 U/mL
CA-125	691.5 U/mL	CA-125	3728.4 U/mL
G-CSF	85.1 pg/mL	PTH-rP	13.7 pmol/L
		PTH	11 pg/mL

WBC, white blood cells; RBC, red blood cells; Hb, hemoglobin; Ht, hematocrit; Plt, platelet count; APTT, activated partial thromboplastin time; PT, prothrombin time; CEA, carcinoembryonic antigen; CA, cancer antigen; G-CSF, granulocyte colony-stimulating factor; TP, total protein; Alb, albumin; T-bil, total bilirubin; AST, aspartate aminotransferase; ALT, alanine aminotransferase; LDH, lactate dehydrogenase; ALP, alkaline phosphatase; ChE, cholinesterase; Glu, glucose; CRP, C-reactive protein; T-chol, total cholesterol; TG, triglycerides; BUN, blood urea nitrogen; Cre, creatine; UA, uric acid; Na, sodium; K, potassium; Cl, chloride; Ca, calcium; P, phosphorus; PTH-rP, parathyroid hormone-related peptide; PTH, parathyroid hormone.
